# Endobronchial Schwannoma Presenting as Persistent Cough: Diagnosis and Successful Bronchoscopic Resection

**DOI:** 10.7759/cureus.107950

**Published:** 2026-04-29

**Authors:** Javier W Torres, Victor Soto, Ricardo Fernandez-Gonzalez, Christian Castillo-Latorre, David Abia-Trujillo, Mariela Ginés-Rosario, Belissa Lopez-Pena

**Affiliations:** 1 Pulmonary and Critical Care Medicine, San Juan City Hospital, San Juan, PRI; 2 Pulmonary and Critical Care Medicine, Hospital Auxilio Mutuo, San Juan, PRI; 3 Pulmonary and Critical Care Medicine, Mayo Clinic, Jacksonville, USA; 4 Internal Medicine, San Juan City Hospital, San Juan, PRI

**Keywords:** benign tumors, debulking procedures, endobronchial lesion, flexible bronchoscopy, lung tumors, schwannomas, transbronchial biopsies

## Abstract

Schwannomas are benign, slow-growing tumors that arise from Schwann cells of the peripheral nerve sheath. Pulmonary schwannomas are extremely uncommon, accounting for approximately 0.2% of all lung tumors and representing a small fraction of tracheobronchial neoplasms. We present a case involving a 49-year-old non-smoking male with a history of Crohn’s disease who reported a persistent productive cough, dyspnea, and a subjective sensation of airway obstruction that did not improve with nebulized therapy. Chest CT revealed an endobronchial lesion obstructing the right mainstem bronchus. Flexible bronchoscopy demonstrated a vascular, pedunculated mass occupying approximately 80% of the bronchial lumen, and transbronchial biopsies were obtained. Histopathological and immunohistochemical analysis confirmed a diagnosis of schwannoma, with strong S100 positivity. The patient subsequently underwent rigid bronchoscopy with argon plasma coagulation, followed by complete tumor resection using a cryoprobe, resulting in successful airway restoration. This report highlights the importance of considering rare benign neoplasms in patients with persistent respiratory symptoms and emphasizes the role of bronchoscopic intervention in both diagnosis and definitive treatment.

## Introduction

Schwannomas, also known as neurilemomas or neurinomas, are benign tumors that arise from Schwann cells of the peripheral nerve sheath. These tumors are typically slow growing, well-circumscribed, and noninfiltrative, and they most commonly develop along cranial or peripheral nerves [1-5]. Although schwannomas can develop in various anatomical locations, pulmonary schwannomas are an exceptionally rare entity, representing approximately 0.2% of all lung tumors and constituting a small fraction of tracheobronchial neoplasms [1,6]. Pulmonary schwannomas may present as intraparenchymal or endobronchial lesions, often presenting with nonspecific respiratory symptoms such as cough, dyspnea, or chest discomfort [2,3]. Due to their rarity and nonspecific clinical presentation, these tumors are frequently misdiagnosed or diagnosed at a late stage in the disease course.

There is limited literature describing pulmonary schwannomas, an entity that accounts for an extremely small proportion of thoracic neoplasms. The rarity of these tumors, combined with their nonspecific clinical presentation, poses a significant diagnostic challenge in routine clinical practice. As such, each well-documented case provides valuable incremental insight into improving recognition patterns, refining differential diagnoses, and guiding appropriate diagnostic strategies.

This case was previously presented as a rapid-fire oral presentation at the American College of Chest Physicians (CHEST) Annual Meeting on October 7, 2024.

## Case presentation

**Figure 1:**
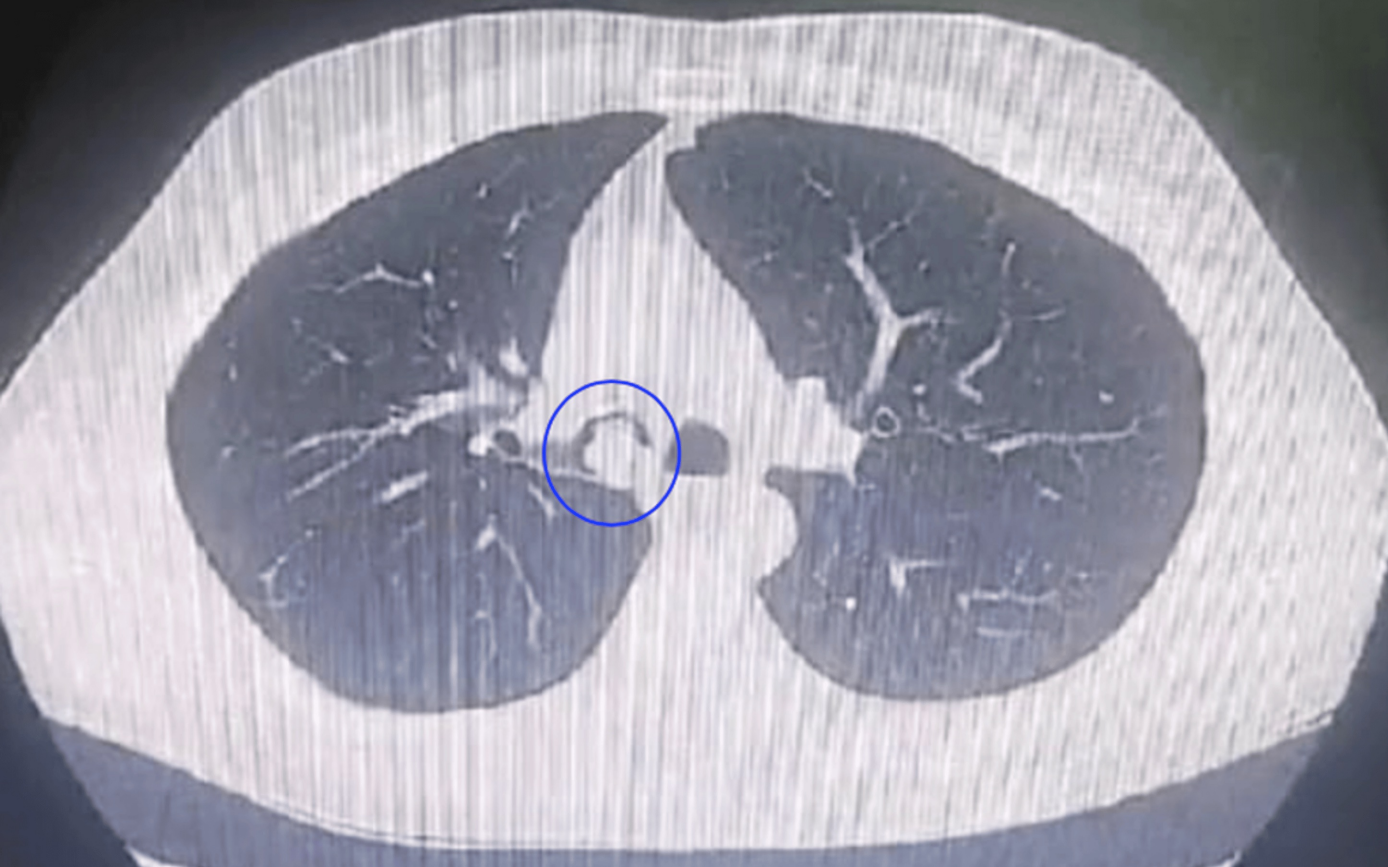
Cross-sectional CT image of the chest showing a round mass (blue circle) in the right main bronchus, measuring 15 × 14 × 14 mm The blue circle delineates the location of the lesion within the right main bronchus. The mass is well-circumscribed and demonstrates a rounded morphology, measuring approximately 15 × 14 × 14 mm CT: computed tomography

A 49-year-old male with a past medical history of Crohn’s disease was referred for evaluation of a persistent cough. The patient was a non-smoker and reported a productive cough unresponsive to nebulized therapy, accompanied by dyspnea and a subjective sensation of airway obstruction, described as a “drowning” feeling. He denied fever, hemoptysis, chest pain, or weight loss. Physical examination was unremarkable, with normal vital signs and oxygen saturation on room air. A chest CT scan revealed a well-defined lesion in the right mainstem bronchus measuring 15 × 14 × 14 mm, causing partial airway obstruction (Figure 1). These findings prompted further diagnostic evaluation.

**Figure 2:**
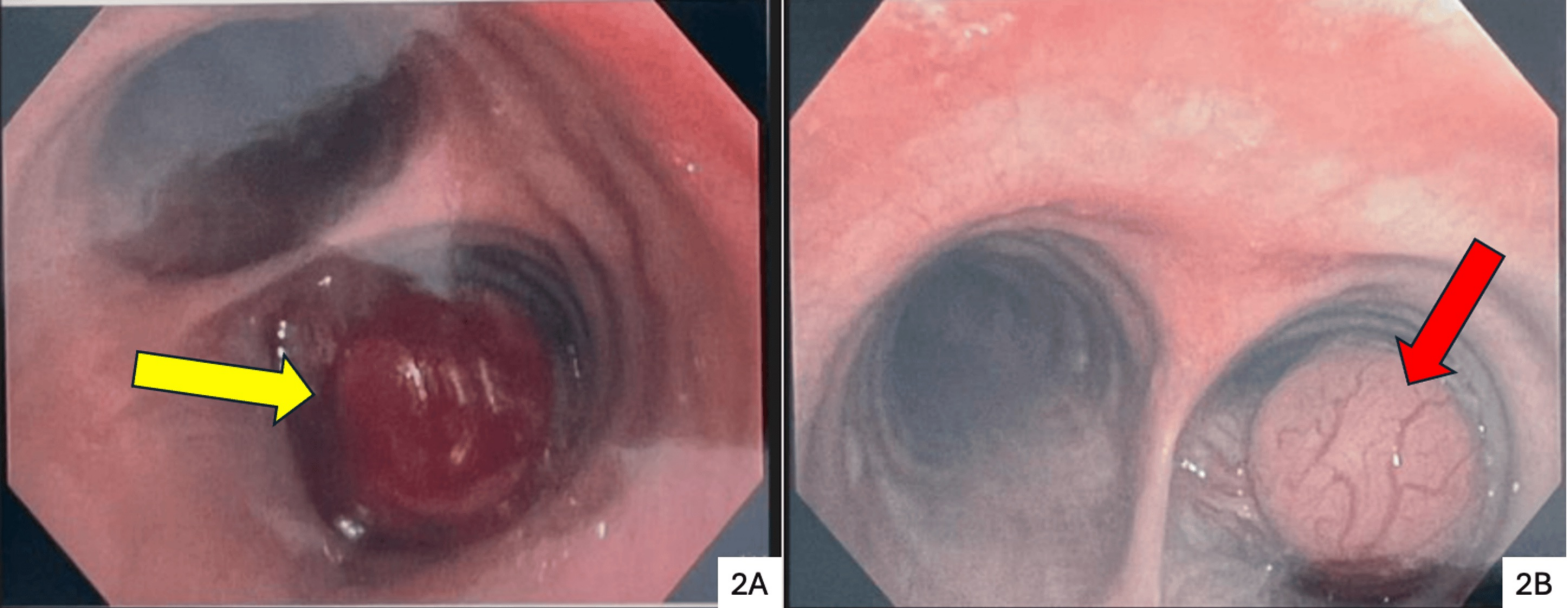
Flexible bronchoscopy findings The right image shows a vascular, pedunculated mass with smooth borders (red arrow) occupying 80% of the right main bronchial lumen (2B). Left image (2A) shows the mass (yellow arrow) after five forceps biopsies

**Figure 3:**
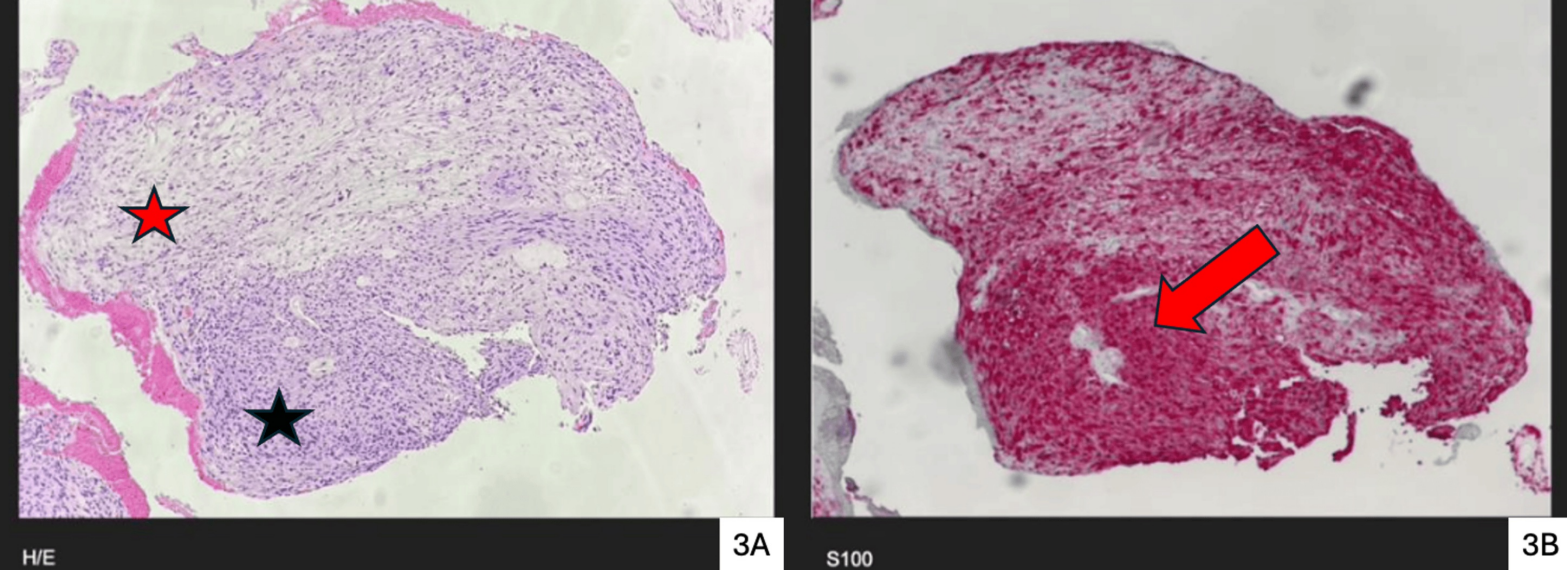
Histology and immunohistochemistry findings Histology of the right main bronchus mass (3A) showing biphasic cellularity (black star: hypercellular areas (Antoni A), red star: hypocellular areas (Antoni B)). Immunohistochemistry (3B) showing strong and diffuse positivity (red arrow) for S-100

Flexible bronchoscopy demonstrated a vascular, pedunculated mass with smooth borders, occupying approximately 80% of the right main bronchial lumen (Figure 2). Five forceps biopsies were obtained without complication, and the patient was discharged the same day. Histopathological examination of the biopsy specimens demonstrated features consistent with schwannoma (Figure 3). Immunohistochemical analysis confirmed the diagnosis, showing strong and diffuse positivity for S-100 protein (Table 1). Tumor cells were additionally focal and/or weakly positive for actin, BCL2, CD99, TTF-1, and vimentin, and negative for ALK-1, CD34, CD45, CK8/18, c-Kit, CK-pankeratin, and EMA (Table 1). The proliferation index was low (<5%), supporting a benign etiology.

**Table 1:** Immunohistochemical profile of the tumor IHC: immunohistochemistry; EMA: epithelial membrane antigen; CK: cytokeratin; ALK: anaplastic lymphoma kinase; BCL2: B-cell lymphoma 2; TTF-1: thyroid transcription factor-1

Marker	Result
S-100	Strong diffuse positive
Actin	Focal/weak positive
BCL2	Focal/weak positive
CD99	Focal/weak positive
TTF-1	Focal/weak positive
Vimentin	Positive
ALK-1	Negative
CD34	Negative
CD45	Negative
CK8/18	Negative
c-Kit	Negative
CK-Pankeratin	Negative
EMA	Negative

**Figure 4:**
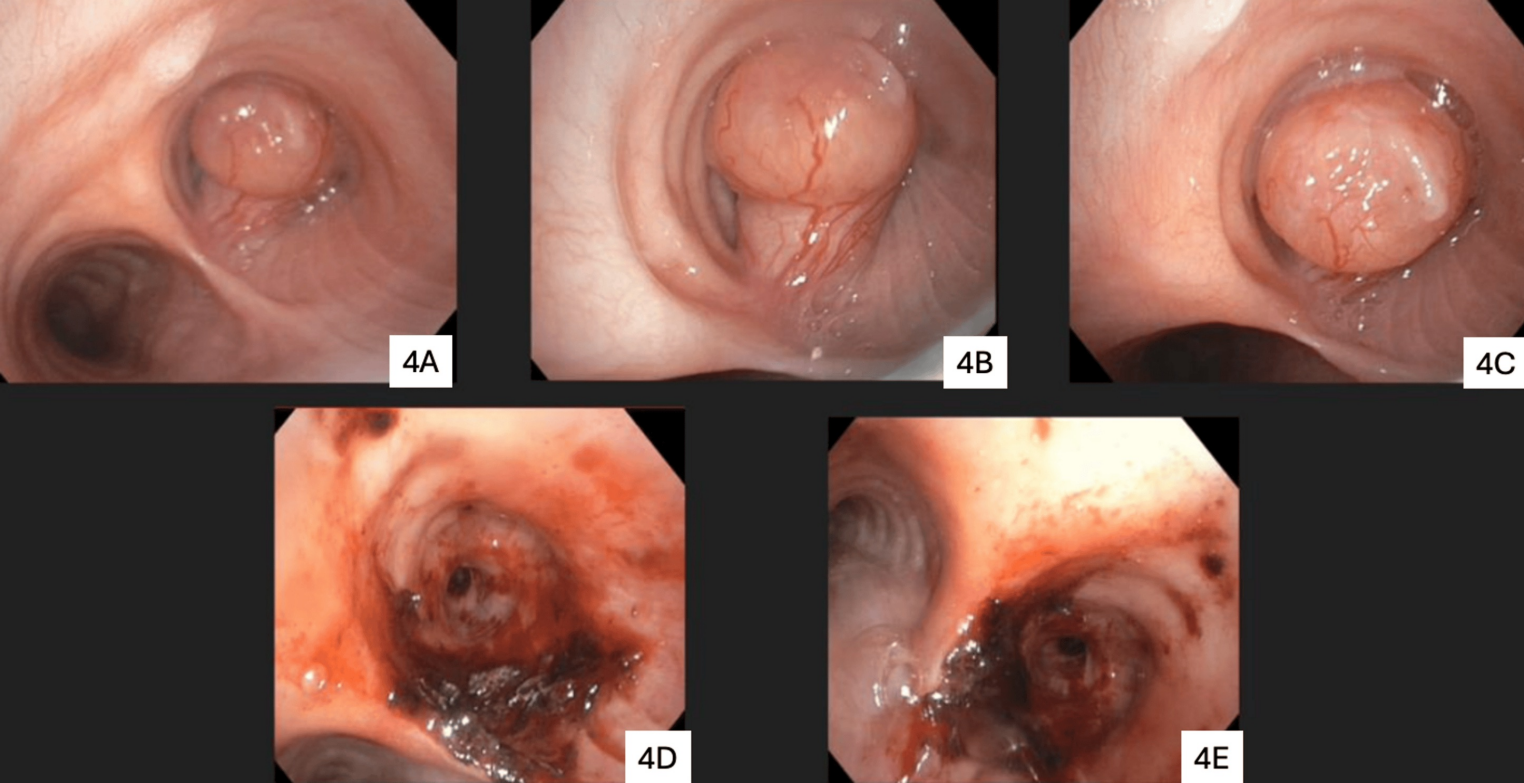
Rigid bronchoscopy findings The images show the vascular, pedunculated mass in the right main bronchus (4A, 4B, and 4C) and post-resection view (4D and 4E)

The patient was subsequently referred to interventional pulmonology for definitive management. He underwent rigid bronchoscopy with argon plasma coagulation followed by tumor debulking and complete resection using a cryoprobe (Figure 4). The procedure was well-tolerated without any complications. Following the intervention, the patient experienced significant improvement in respiratory symptoms, with resolution of cough and dyspnea. He has remained under clinical follow-up for the last eight months, with no evidence of recurrence to date. Although recurrence after complete resection of schwannomas is rare, continued surveillance is planned.

## Discussion

Schwannomas are benign peripheral nerve sheath tumors that may rarely arise within the thoracic cavity. Pulmonary involvement is exceedingly uncommon, accounting for approximately 0.2% of lung neoplasms and a small proportion of tracheobronchial tumors, which contributes to diagnostic difficulty [1,6]. Although frequently asymptomatic, these lesions may present with nonspecific respiratory symptoms such as cough, dyspnea, or wheezing, often mimicking more common airway conditions and delaying recognition [7]. Imaging typically reveals a well-circumscribed lesion, while flexible bronchoscopy remains essential for direct visualization and tissue sampling [1,4]. In this patient, CT and bronchoscopy identified a large endobronchial mass causing significant airway obstruction, emphasizing the need for early evaluation in patients with persistent or refractory respiratory symptoms.

Histopathological evaluation of the biopsy specimens revealed a well-circumscribed spindle cell neoplasm composed of elongated cells with tapered nuclei and indistinct cytoplasmic borders, arranged in interlacing fascicles. Areas suggestive of alternating cellularity (Antoni A and Antoni B patterns) were appreciated (Figure 3), with focal nuclear palisading consistent with Verocay body formation, supporting a neural sheath origin [5]. No significant cytologic atypia, necrosis, or increased mitotic activity was identified.

Immunohistochemical profiling further substantiated the diagnosis of schwannoma. The tumor demonstrated strong and diffuse positivity for S-100 protein, a hallmark of Schwann cell differentiation, confirming a neural crest origin [5]. Additional staining showed focal and/or weak positivity for actin, BCL2, CD99, TTF-1, and vimentin (Table 1). While vimentin supports mesenchymal lineage, the limited and focal expression of actin and CD99 is nonspecific and may be seen in a variety of soft tissue tumors. The weak TTF-1 expression should be interpreted with caution, as it is not characteristic of schwannomas and may represent nonspecific staining or background reactivity.

Importantly, the tumor was negative for a broad panel of markers that help exclude key differential diagnoses. The lack of cytokeratin expression (CK8/18, CK-pankeratin) and EMA argues against epithelial or perineurial tumors. Negative CD34 reduces the likelihood of a solitary fibrous tumor, while the absence of CD45 excludes a lymphoproliferative process. Negative c-Kit (CD117) makes gastrointestinal stromal tumor unlikely, and the lack of ALK-1 expression argues against an inflammatory myofibroblastic tumor. The proliferation index (Ki-67) was low (<5%), consistent with an indolent growth pattern and supporting a benign lesion. Taken together, the morphologic features and immunoprofile are diagnostic of a benign schwannoma, with no evidence of malignant transformation.

Although generally benign, endobronchial schwannomas can cause clinically significant airway compromise. Management is focused on complete resection. Bronchoscopic techniques, including argon plasma coagulation and cryotherapy, provide an effective and minimally invasive approach for selected lesions, allowing both diagnosis and treatment with favorable outcomes and low recurrence rates [1,7]. Recognition of this entity is essential in patients with persistent respiratory symptoms, as timely bronchoscopic evaluation enables definitive diagnosis and appropriate management [7].

From a differential diagnosis standpoint, endobronchial schwannomas must be distinguished from a broad spectrum of benign and malignant airway lesions. Benign considerations include hamartomas, papillomas, lipomas, and inflammatory polyps. Malignant etiologies include carcinoid tumors, mucoepidermoid carcinoma, adenoid cystic carcinoma, and primary bronchogenic carcinoma. Radiographically, the presence of a well-defined, noninvasive lesion favors a benign process. However, definitive differentiation relies on histopathology and immunohistochemistry, particularly strong S-100 positivity and absence of epithelial markers, which help exclude carcinomas.

Management is guided by tumor size, location, symptom burden, and degree of airway compromise. The primary goal remains complete resection while preserving lung parenchyma whenever feasible. Despite their benign histology, complications can be clinically significant and are primarily related to mechanical airway obstruction. Progressive luminal narrowing may result in recurrent post-obstructive pneumonia, atelectasis, air trapping, or bronchiectasis in the affected segment. In severe cases, acute airway compromise can occur, particularly if there is rapid growth or superimposed inflammation. Hemoptysis, although uncommon, may be seen due to mucosal irritation or tumor vascularity. Rarely, malignant transformation has been reported but remains exceedingly uncommon. Procedure-related complications should also be considered, including bleeding, airway perforation, or post-intervention stenosis following bronchoscopic or surgical management.

Histopathological examination remains the diagnostic cornerstone, demonstrating the characteristic biphasic Antoni A (hypercellular) and Antoni B (hypocellular) architecture, with occasional Verocay bodies. Immunohistochemically, strong and diffuse S-100 protein positivity confirms Schwann cell origin, while low proliferative indices (e.g., Ki-67 <5%) further support benign behavior, as observed in this case.

## Conclusions

While pulmonary schwannomas are rare, they should be included in the differential diagnosis of persistent or unexplained endobronchial lesions. A high index of suspicion, coupled with timely imaging and bronchoscopic evaluation, is essential to avoid delays in diagnosis. Early recognition enables minimally invasive management and prevents complications related to chronic airway obstruction.
